# The neuroanatomy of speech sequencing at the syllable level

**DOI:** 10.1371/journal.pone.0196381

**Published:** 2018-10-09

**Authors:** Feng Rong, A. Lisette Isenberg, Erica Sun, Gregory Hickok

**Affiliations:** Department of Cognitive Sciences, University of California, Irvine, Irvine, CA, United States of America; Baycrest Health Sciences, CANADA

## Abstract

Correctly ordering a sequence of speech sounds is a crucial aspect of speech production. Although studies have yielded a rich body of data on the neural substrates of visuomotor sequencing and sequence learning, research on brain regions and their functions involving speech sequence production hasn’t attracted much attention until recently. Previous functional MRI studies manipulating the complexity of sequences at the phonemic, syllabic, and suprasyllabic levels have revealed a network of motor-related cortical and sub-cortical speech regions. In this study, we directly compared human brain activity measured with functional MRI during processing of a sequence of syllables compared with the same syllables processed individually. Among a network of regions independently identified as being part of the sensorimotor circuits for speech production, only the left posterior inferior frontal gyrus (pars opercularis, lIFG), the supplementary motor area (SMA), and the left inferior parietal lobe (lIPL) responded more during the production of syllable sequences compared to producing the same syllables articulated one at a time.

## Introduction

Fluent speech requires the rapid coordination of vocal tract gestures to produce the intended sequence of phonemes (segments), syllables, and words. Speech error data (slips of the tongue, Spoonerisms) have illuminated the process, providing direct evidence for sequence planning over multiple representational levels [1–5]. The following examples illustrate sequence errors at the phoneme, syllable, and word levels [5] (*intended utterance → actual (slip) utterance*):

- phoneme: *keep a tape→teep a kape*
- syllable: *philosophy→phi-so-lo-phy; butterfly and caterpillar→butterpillar and caterfly*
- word: *a computer in our own laboratory→a laboratory in our own computer*

Such evidence from natural slips of the tongue, laboratory induced slips [6], chronometric studies of object naming [7], computational modeling [8], and speech error data following brain injury [9, 10] has led to much progress in understanding the cognitive mechanisms behind the sequencing of speech sounds during language production [2, 3, 8, 9, 11].

A number of studies have investigated the neural foundation of speech production using a range of methods with notable progress in mapping the broad stages of speech (e.g., lexical-semantic versus phonological) onto neural networks [12–17]. Another line of research has made significant progress in understanding the role of sensorimotor circuits in speech motor control [10, 18–24]. Overall these studies have identified a distributed speech production network that includes pre- and post-central gyri, medial premotor cortex (SMA/pre-SMA), lateral premotor cortex, posterior inferior frontal gyrus, anterior insula, superior temporal gyrus, and the posterior planum temporale region, termed Spt [25, 26], as well as portions of the cerebellum and basal ganglia [24].

Relatively few studies, however, have explicitly studied the neural circuits that support speech sequencing, an endeavor that could eventually link psycholinguistic and neural models of speech planning. One functional MRI study that did so [27] manipulated sequence complexity (number of unique syllables in the set: *ta-ta-ta* vs. *ka-ru-ti*) and reported activations associated with greater complexity in a network including pre-SMA, frontal operculum/anterior insula (bilaterally), lateral premotor cortex and the posterior inferior frontal gyrus/sulcus (left lateralized). One limitation of this study, however, is that it is impossible to know whether the activations are driven by the sequencing demands *per se* or simply by the increased complexity demands associated with articulating different tokens, independently of whether the sequence is correctly produced. For example, repeating the same token three times versus three different tokens should lead to a difference in the degree of neural adaptation in regions coding motor plans for syllables independently of the sequencing demands. This study, along with others (Shuster & Lemieux, 2005), also manipulated syllable complexity (number of segments within the syllable), which should increase segment sequencing load; a similar network was implicated. But again, because syllable complexity manipulations involve a different number of phonemes produced, it cannot definitively isolate sequencing *per se* as opposed to simply more time spent planning articulation.

One fMRI study [16] moved toward avoiding these confounds by contrasting several articulatory conditions, one in which the same item was produced repeatedly (PIGRA PIGRA PIGRA PIGRA…), a second with two alternating items using the same phonemes but with reordered syllables (ZE.KLO KLO.ZE ZE.KLO KLO.ZE…), a third with two alternating items using the same phonemes but in two different syllabification patterns (LO.FUB FU.BLO LO.FUB FU.BLO…), and a fourth with variable phonemes and syllables (GUPRI DRAVO VIBAG NUVAF…). Activation patterns to the various conditions differentiated some of the previously identified regions involved in syllable complexity/sequencing. Specifically, the left SMA responded similarly to the first three conditions and greater to the fourth (variable) condition; the ventral premotor cortex responded similarly to the first two conditions, greater to the third (alternating syllabification pattern) condition, and most to the fourth (variable) condition; and the right cerebellum responded least to the first condition, more to the second (alternating syllable order) and third (alternating syllabification pattern) conditions, which did not differ from one another, and most of the fourth (variable) condition. The authors concluded that (i) the left SMA codes speech information at the phoneme level, thus maximally activating only when there is variation in the articulated phonemes, (ii) the left ventral premotor cortex codes speech information at the syllable level, thus modulating activity as a function of variation in the syllable structure, and (iii) the right cerebellum is coding speech information at the suprasyllabic level, thus modulating activity as a function of variation in syllable order (or structure). This finding and interpretation challenges the idea that the ventral premotor cortex and SMA play a particular role in syllable sequencing. However, as this study involved overt speaking, which is known to modulate activity in motor related areas [20, 25, 28], it is unclear whether the different activity profiles are influenced more by response properties during speech planning or a mixture of planning and perception, which could vary from one region to the next (although [27] also used overt speech).

Yet another approach to mapping the network involved in speech sequence processing is to identify regions involved in learning novel sequences. Segawa and colleagues [29] compared fMRI activation patterns to novel versus previously learned non-native (phonotactically illegal) phoneme sequences and reported greater activation for novel sequences in premotor cortex, including both dorsal and ventral (par opercularis) clusters, the frontal operculum (FO), the superior parietal lobule, posterior superior temporal gyrus (pSTG) and posterior superior temporal sulcus (pSTS), inferior temporal-occipital cortex, and globus pallidus. One complication in interpreting these results is that the duration spent articulating learned and novel sequences differed, making it hard to attribute to activation differences to the sequence processing. An additional analysis showed that the frontal operculum activity was significantly correlated with learning success, which alleviates this concern, and suggests that the FO may play an important role in speech sequencing.

The present study sought to map the network involved in speech sequence planning without the potential confounds of overt sensory responses, while taking a different approach to avoiding confounds associated with differing numbers of syllable types or phonemes in the contrasting conditions. Our approach involved the auditory presentation of four different syllables for both the sequence and the non-sequence conditions. In the sequence conditions, the four syllables were presented immediately one after the other, after which the participant covertly repeated the entire sequence. In the non-sequence (“unit”) condition, a short silent interval occurred after each syllable was presented, during which the participant silently repeated that one syllable. Thus, on each 4s trial in each condition the same set of syllables was planned and covertly repeated but differed in terms of whether each syllable was repeated individually or as a sequence. Activations under these conditions were examined in the context of a set of ROIs defined by an independent localizer designed to elicit activations associated with listening to and covertly repeating syllables generally. This method allowed us to identify distinct sub-networks involved in generating individual syllables versus syllable sequences.

Before turning to the experiment, a brief comment is warranted on what precisely we mean here by “sequencing.” From a linguistic perspective, most models assume a distinction between frame and content, where a higher-order frame serves as a planning unit into which phonological content is inserted [1, 2, 4, 5, 8, 30]. Thus, sequencing is achieved using hierarchical coding. Similar ideas regarding hierarchical coding of action sequences have emerged in non-linguistic motor control research where the computational motivation is that hierarchical control is a way to manage combinatorial explosion given the degrees of freedom available for achieving a motor goal (the motor equivalence problem [31]) and which is exacerbated in a sequence of actions [32–34]. In the present paradigm, however, it is unlikely that a sequence of randomly ordered syllables will be instantaneously coded as a word- or phrase-like chunk under a higher-level node, as chunking requires some amount of learning [35]. Instead, our paradigm more likely taps into processes involved in temporarily storing and/or modifying a sequence plan, i.e., some form of phonological working memory [36, 37]. Guenther has proposed phonological working memory plays a key role in speech production and that the underlying representation is roughly a syllable-size unit comprised of onset, nucleus, and coda slots [24]. According to this formulation, sequences of syllables are coded using a gradient of activation over the planned units with the most active unit being produced first and so on. Although the present study will not clarify the nature of the representations or computations, it will help clarify the neural networks involved.

## Materials and methods

### Participants

Nineteen healthy adults (ten female) between 18 and 35 years of age participated in the study. All volunteers were right-handed native English speakers with normal or corrected-to-normal vision, self-reported normal hearing, no known neurological disease, and no other health situations that would preclude participating in MRI experiments. Informed consent was secured prior to the study, which was approved by the Institutional Review Board in University of California, Irvine. Two participants failed to complete the two-session protocol because of personal non-health related reasons.

### Stimuli and tasks

The auditory stimulus pool was composed of six 500-millisecond audio clips (Audacity 1.2.5, 44.1 kHz, 32-bit) recorded by a male native English speaker in a sound attenuated room. Each audio clip consisted of one consonant-vowel (CV) syllable (‘ba’, ‘da’, ‘ka’, ‘ga’, ‘ta’, or ‘pa’), recorded at a natural speed. These stimuli were used in all conditions described below.

**Fig 1:**
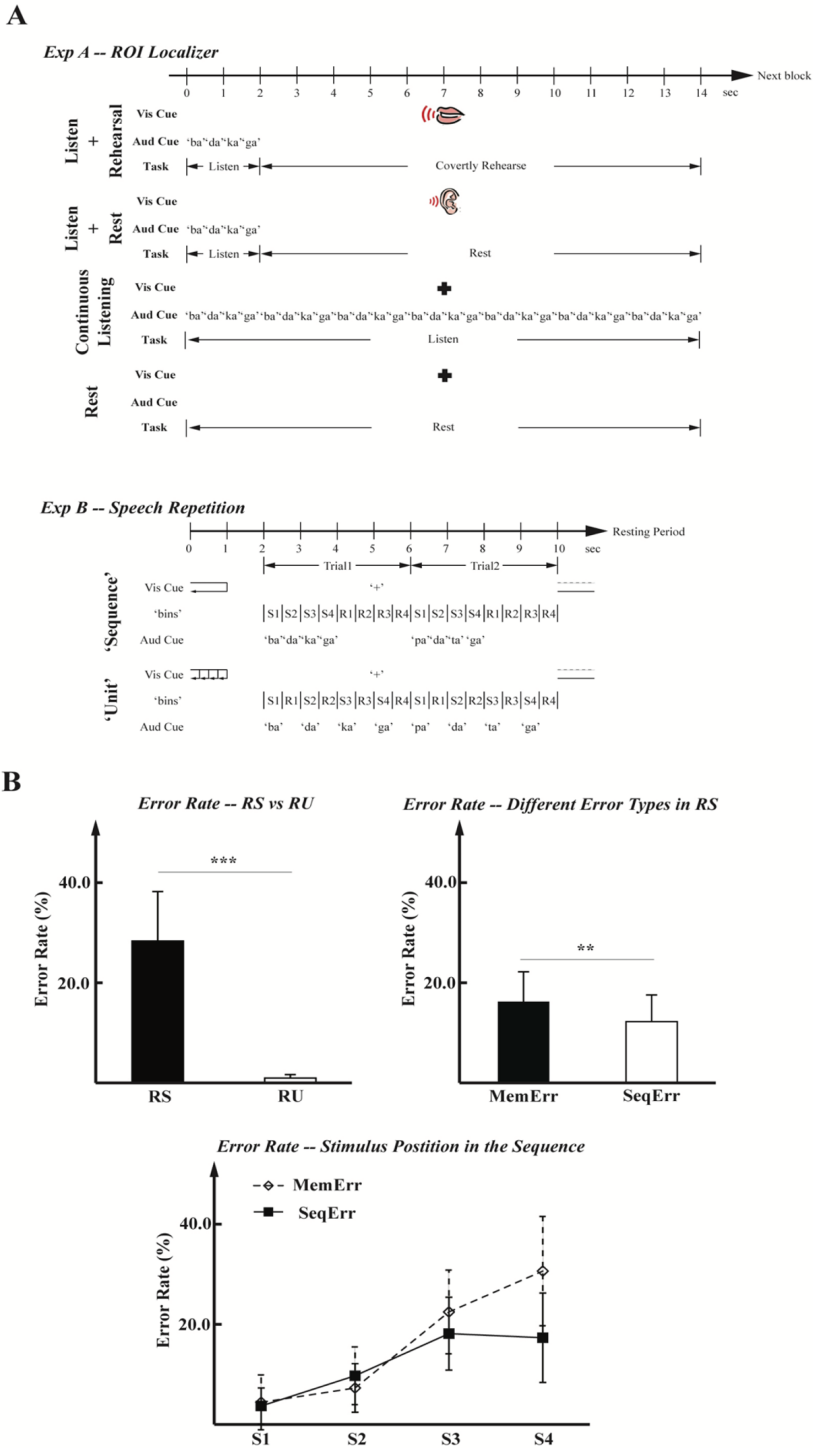
Experimental tasks and behavioral results. (A) The visual cue, auditory cue, and task timelines for ‘ROI Localizer’ and ‘Speech Repetition’ tasks. From top to bottom: ‘ROI Localizer’ tasks included ‘Listen + Rehearsal’, ‘Listen + Rest’, ‘Continuous listen’, and ‘Resting’ conditions. Each block had one trial of one task with 14 seconds duration. ‘Speech Repetition’ tasks included ‘Repeat Sequences (RS)’ and ‘Repeat Units (RU)’ conditions. Each block had two consecutive trials of one task. Each trial was 4 seconds long. ‘S1’ through ‘S4’ represented the auditory CV syllable cues in their orders within each trial, and ‘R1’ through ‘R4’ represented the required responses or repetition of the cues in their corresponding order. (B) The group level results from the ‘Speech Repetition’ tasks in behavior sessions, in which all repetitions were made overtly. The top figures depicted mean error rates over different task types (‘RS’ and ‘RU’), and mean error rates over different error types (‘SMErr’–substitution and missing; ‘SeqErr’–sequencing error) within the RS condition, respectively. The lower figure showed mean error rates for each syllable in the sequence (S1 –S4) during performing the RS task. The substitution and missing error rates were plotted in diamond signs and dash lines; the sequencing error rates were plotted in square signs and solid lines. The error bars represented the standard error of means (SEM).

*Repetition tasks*. The primary aim of the study was to identify sub-components of the speech auditory-motor network that are differentially involved in the production of individual syllables versus syllable sequences. We therefore designed two different covert repetition tasks: one condition involved the covert (imagined) repetition of individual syllables and the other involved the covert repetition of syllable sequences. The reason for using covert speech was to avoid auditory activation due to perception of self-produced vocalization. To control for simple duration effects, we utilized trials of equal duration (4s) and an equivalent number of syllables (4) for each repetition condition; the trials differed only in whether the subject repeated each heard syllable one-at-a-time or as a sequence of four syllables (Fig 1A). Specifically, in a Repeat Unit (RU) condition trial, 4 different auditory syllable stimuli were presented at a rate of 1Hz and participants repeated each syllable immediately after hearing it during the 500 millisecond inter-stimulus interval. In the Repeat Sequence (RS) condition, 4 different auditory syllable stimuli were presented at a rate of 2Hz in the first half of the trial and participants repeated the entire sequence in the second half of the trial. Thus, for both conditions within each 4s trial participants heard and generated 4 syllables. The conditions were grouped into 10s activation blocks that contained two 4s trials of the same condition and were preceded by a 1s visual instruction indicating the type of task (RU or RS, Fig 1A) and a 1sec silent period. Activation blocks alternated with 10s silent “resting” blocks, during which the participant maintained fixation on a “+” symbol.

*Localizer scans*. In addition to the repetition tasks described above, we also included localizer scans to identify networks in each participant that are involved in the perception and production of speech. The logic behind using a localizer is to precisely define the relevant network, thus (i) minimizing the possibility of spurious activation, (ii) increasing statistical sensitivity and our ability to evaluate null results, and (iii) minimizing possible condition differences due to executive control and related mechanisms. Our speech processing localizer scans were modeled on a previous study (Hickok et al. 2009). It consisted of four task conditions using the syllable stimuli mentioned in the above covert repetition tasks: (1) listen + rehearsal; (2) listen + rest; (3) continuous listen; and (4) rest. Tasks were presented in pseudorandom order, such that the same condition never occurred twice in a row. For this set of tasks, each block was 14 seconds in duration. For each of the listen + rehearsal and listen + rest blocks, a four-CV-syllable stimulus was presented during the first two seconds, the same as the stimulus presentation in the RS condition, and the participants either rehearsed the set repeatedly or rested for the next 12 seconds until the onset of the next trial. For each of the continuous listen blocks, the same syllable sequence was presented to the participant auditorily seven times throughout the 14 second duration without any response being required from the participant. Each resting block had a 14 second silent period. A visual cue was presented during the full length of each block to inform the participant of the experimental condition (Fig 1A). We note that the localizer task includes the sequence (RS) condition used in the main experiment. This is by design as we aimed to identify the speech network with the most difficult condition and then independently determine which of the ROIs were modulated by the sequence versus non-sequence manipulation.

For each participant, a behavioral session preceded the functional imaging session. The behavioral session served both as training and as a means to measure individual variability in performance in our subject sample. From a task standpoint, the behavioral and functional imaging sessions differed only in the manner of overt (behavioral session) versus covert (functional imaging session) repetition or rehearsal of the auditory cues. Each session had 4 ‘repetition’ runs and 3 ‘localizer’ runs. Each ‘repetition’ run had 18 pseudo-randomized blocks (9 for each condition). Each localizer run had 20 pseudo-randomized blocks (5 for each condition). Before the behavioral session, 3–4 practice runs with the same tasks and stimuli from the same pool were given to each participant to guarantee he/she had learned the tasks and was familiar with the stimuli so that his/her performance had reached a steady state. For both sessions, the stimuli were presented through headsets. In the behavioral session, an AKG HSC271 professional headset (AKG Acoustics, Vienna, Austria) was used for both acoustic presentation and vocal response recording. The recorded overt responses were analyzed later to assess the participant’s performance. In the MRI session, an MR compatible Res Tech headset was used for delivering the auditory stimuli. Stimulus presentation and timing control were realized using Cogent software (http://www.vislab.ucl.ac.uk/cogent_2000.php) implemented in Matlab 7 (Mathworks, Inc., USA).

### MRI scanning parameters

We acquired the functional MR images with a Philips Achieva 3T scanner (Philips Healthcare, Andover, MA) equipped with an 8-channel SENSE receiver coil at the University of California, Irvine Research Imaging Center facility. Using a gradient echo EPI pulse sequence, we collected a total of 1120 EPI volumes over the seven runs (TR = 2000 ms, TE = 26 ms, flip angle = 70°, FOV = 220 x 220 x 128 mm^3^, within-plane matrix = 112 x 112, voxel size = 1.964 x 1.964 x 3.0 mm^3^) for each participant. Thirty-two sequentially acquired axial slices covered the whole brain with a 3 mm depth and 1 mm gap per slice. In addition to the functional scans, we also acquired a high-resolution T1-weighted anatomical image with a magnetization prepared rapid acquisition gradient echo (MPRAGE) pulse sequence (TR = 11 ms, TE = 3.55 ms, FOV = 240 x 240 x 150 mm^3^, 150 axial slices with 1 mm depth each, within-plane matrix = 240 x 240, voxel size = 1 x 1 x 1 mm^3^) for each participant.

### Data analysis

#### Behavioral data analysis

During the behavioral session, the participant’s vocal responses in the repetition tasks were recorded using Audacity (Audacity 1.2.5, 44.1 kHz, 32-bit) and compared to the cues. In this study, we used the repetition error rates to assess each participant’s performance. For each trial, two types of behavioral errors were identified: the first type involved errors on individual syllables, which were evident either in the form of omissions or substitutions. For this type of error the participant either failed to repeat the syllable (omission) or repeated a syllable that was not cued (substitution). The second type involved sequencing errors, which applied to the RS condition only, and were evident in cases where the participant repeated all four syllables in the cued sequence but in an incorrect order. For the RS condition, if a syllable was repeated twice, the out-of-place one was counted as a substitution error. Error rates for each error type were calculated for each participant as the ratio of incorrectly repeated syllables over the total number of syllables, and were computed for each ‘repetition’ run and averaged across the four runs to compare the participant’s performance under RS and RU conditions. In the RS condition, we additionally calculated error rates of each type and the syllable’s place in the sequence (the first, second, third and fourth syllable). For comparison between the RU and RS conditions, the variances were analyzed using one-way repeated-measures ANOVA with Greenhouse-Geisser correction of sphericity. For the error analysis within RS condition, two-way repeated-measures ANOVA was applied, the factors are error types (with two levels: missing/substitution versus sequencing) and place of order in the sequence (with four levels: first, second, third, and fourth). Further post-hoc contrasts were taken with Tukey Honest Significant Differences (TukeyHSD) comparison.

#### MRI data analysis

The MRI data were first preprocessed with applications provided by AFNI software toolbox (Cox, 1996; http:://afni.nimh.nih.gov). The preprocessing procedures included slice timing correction, spatial alignment, image co-registration, normalization, spatial smoothing, and amplitude scaling. First, an integrative process implemented slice-timing correction and spatial alignment among the functional scans, followed by spatial alignment and co-registration of the EPI images to the anatomical image. The spatial alignment was done using a 6-parameter rigid body model for motion correction. Second, using Advanced Normalization Tools (ANTs; Avants and Gee, 2004; Avants et al., 2008), the motion corrected images were normalized to an EPI template in Montreal Neurological Institute (MNI) space specifically facilitated with anatomical images from current study. This normalization procedure included the following steps: (1) we created a group average anatomical image from the high-resolution anatomical images of all participants using a symmetric diffeomorphic registration method (Avants et al., 2010); (2) we mapped the group anatomical template to MNI space with diffeomorphic transformations provided by ANTs applications; (3) we applied the derived transformation matrices to register the motion corrected functional images to the group average anatomical image and then the general template in MNI space. The third step of preprocessing consisted of spatial smoothing of the normalized images with an isotropic Gaussian kernel of 6mm full width at half maximum (FWHM), high-pass filtering with stop frequency at 0.008 Hz, and scaling relative to the mean.

We then measured the within-individual effect size of the experimental conditions with general linear models specifically constructed for the ‘repetition’ and ‘localizer’ tasks. For the ‘repetition’ runs, 9 regressors were included. In addition to task regressors corresponding to the RS and RU conditions (treated as blocks), another stimulus-timing related regressor (Snd_Event) was used to regress out the impact by the different stimulus presentation frequencies in RU (1Hz) and RS (2Hz), even though the effect of this difference was expected to be negligible at the block level and indeed produced no effect in auditory cortex where the presence of an effect should be most evident. The ‘Snd_Event’ regressor was created by convolving the auditory stimulus presentation-timing vector with a model hemodynamic response function (HRF). Six motion regressors were also included in the linear regression model to address head movements during scanning. We also included one linear contrast (RU vs. RS) in this model. For the ‘localizer’ runs, another linear regression model with three task regressors (listen + rehearsal, listen + rest, and continuous listen) and six motion regressors was applied. In this model we included another linear contrast of (listen + rehearsal vs. listen + rest). For both models, the resulting coefficients for the experimental task conditions represented the mean percent signal change (PSC) values for the conditions as compared to the control (rest); the coefficients for the contrasts represented the mean relative PSC between the contrasted conditions.

For group-level analysis of variance, we input the coefficients and t-scores obtained from individual participants into a mixed-effect and meta-analysis method (3dMEMA in AFNI; Chen et al., 2012), which treated the variability among participants as a random effect. The analysis created a statistical parametric map (SPM, which included coefficients and t-scores) for each of the experimental conditions or contrasts of interest. The significance thresholds were set at p<0.05 after being corrected for family-wise errors (FWE), which were obtained using a Monte Carlo method (3dClustSim in AFNI). The method determines the cluster-size threshold of significance by taking into account the variance among the voxels and the uncorrected statistics of each voxel within the cluster (Forman et al. 1995).

#### Region of interest identification

To assess the functional role of brain regions in sequencing syllables during production, we first defined regions of interest (ROIs) from the localizer scans aimed at identifying regions that were differentially involved in three phases of our task: speech perception, auditory-motor integration, and speech generation. The aim of this process was to identify the broad network involved in the range of processes that contribute to speech production in our task and then to assess which nodes in the network are sensitive to the sequence manipulation. ROIs for auditory speech perception were identified with the contrast ‘continuous listen’ > ‘listen + rehearsal’. This contrast identifies voxels that are responsive to acoustic stimulation as the two conditions differ substantially in the duration of the acoustic event, but factors out motor-related regions. ROIs for generating speech were identified by the contrast ‘listen + rehearse’ > ‘listen + rest’. ROIs for auditory-motor integration were identified by the conjunction of ‘listen + rehearse’ > ‘rest’ (which primarily identified voxels activated during rehearsal) and ‘continuous listen’ > ‘rest’ (which identified voxels activated during listening). For a discussion of the idea that this ROI is involved in an integrative process, see [19, 20, 25, 26]. The responses to the sequence manipulation were examined within these ROIs, and were independent of the ROI identification contrasts. For the auditory-motor processing ROIs, the threshold for ROI identification was set at uncorrected p<0.005 for each voxel and with a conjunction cluster size > 20. For the simple contrasts aimed at identifying speech perception and generation ROIs, the threshold was set at uncorrected p<0.005 for each voxel and corresponding cluster size obtained from the Monte Carlo method so that the FWE corrected p < 0.05. The ROI-based contrasts were statistically measured with paired t-test and the resulting statistics were tested for significance based on the threshold obtained from the above-mentioned Monte Carlo procedures applied to the group-level SPMs for corresponding contrasts.

#### Functional imaging and behavioral results correlation

We reasoned that individual variation in the ability to perform the repetition tasks may be correlated with variation in brain responses. To assess this possibility we computed the correlation between the averaged activity among voxels within each ROI and the averaged error rates from all participants. Similar to statistical analysis of the fMRI data, the significance threshold was set at p<0.05 with FWE correction, based on the Monte-Carlo analysis of the group-level covariate SPM between the functional imaging measurements and the behavior measurements. The covariate SPM was obtained through the ‘-covariate’ option in 3dMEMA process.

## Results

### Behavior

As demonstrated in Fig 1B, the participants made more errors in repeating sequences (Error rate for RS: 28.3% ± 9.89%, mean ± SD) than in repeating individual syllables (Error rate for RU: 0.9% ± 0.69%; F[1,16] = 132.9, p<0.001). The error rate on the RS condition is similar to what is expected in tongue twister covert speech error elicitation paradigms [38]. Under the RS condition, the participants’ error rates demonstrated main effects of error type (F[1,16] = 9.4, p<0.01) and place of order (F[3,48] = 105.9, p<0.001), as well as the interaction (F[3,48] = 15.1, p<0.001) of these two factors. Post-hoc Tukey HSD pair-wise comparison showed they made more omission and substitution errors (error rate: 16.2% ± 5.99%) than sequencing errors (Error rate: 12.2% ± 5.33%; t[16] = 3.05, adjusted p<0.005), mainly due to the higher omission and substitution error rates in repeating the fourth syllable in the sequences (omission and substitution error rate: 30.6%± 10.92%; sequencing error rate: 17.3% ± 8.92%; t[16] = 4.8, adjusted p<0.001). In addition, the participants made significantly more errors in the late elements (the third and fourth syllables) than the early elements (the first and second syllables) for both types of errors. For omission and substitution errors, the averaged early error rate was 5.8% ± 4.76% (mean ± SEM), the averaged late error rate was 26.5% ± 8.91% (t[16] = 11.0, adjusted p<0.001). For sequencing errors, the averaged early error rate was 6.7% ± 4.29%, the averaged late error rate was 17.7% ± 7.50% (t[16] = 7.6, adjusted p<0.001).

### Speech repetition activated ROIs

**Fig 2:**
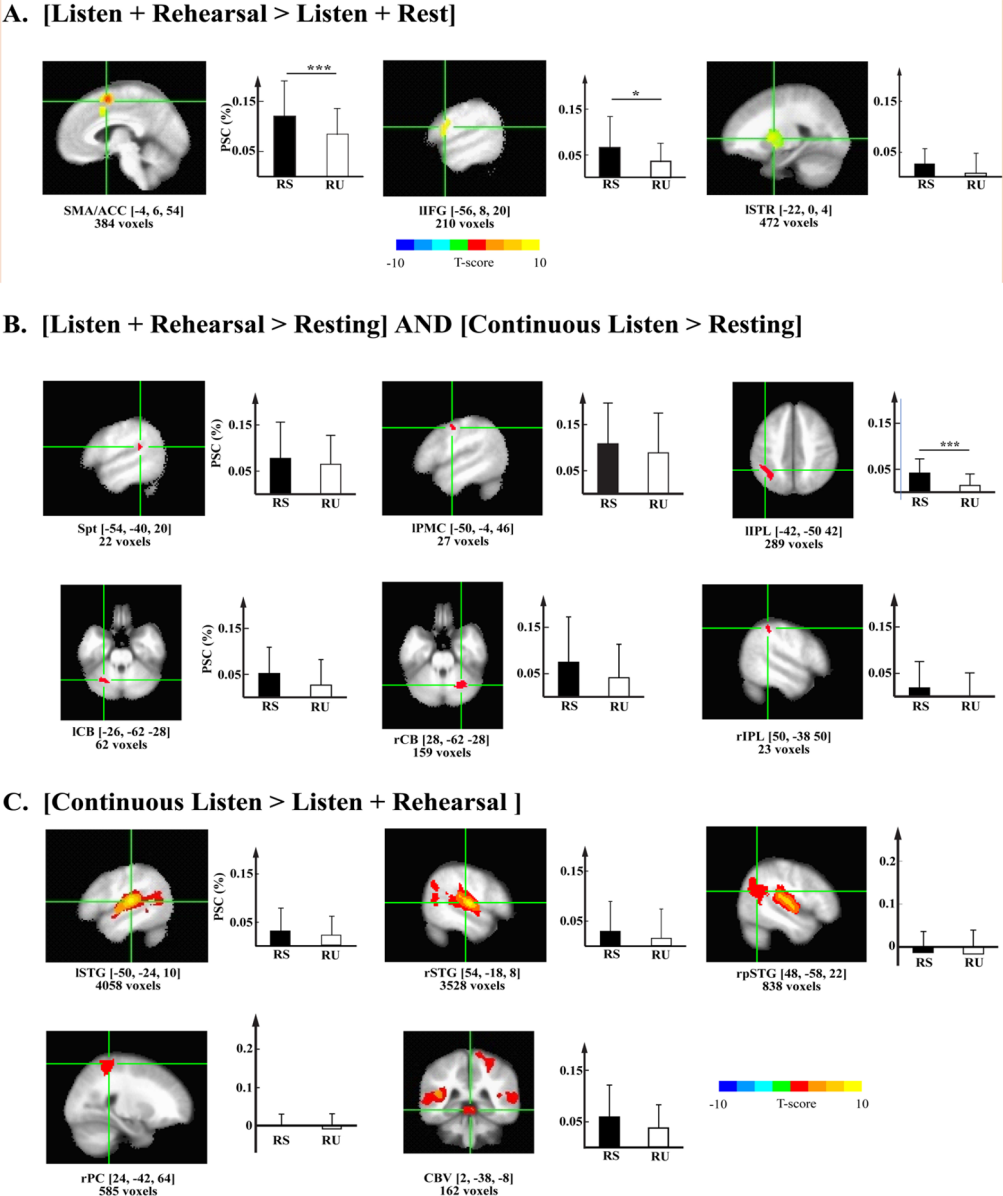
The ROIs and their averaged responses during repetition task performance. (A) The ROIs demonstrated significant stronger activity in ‘Listen + Rehearsal’ condition than ‘Listen + Rest’ condition. From left to right are ROIs with their centers in Supplementary Motor Area (SMA) / Anterior Cingulate Cortex (ACC), left Inferior Frontal Gyrus (lIFG), and left Striatum (lSTR) / Basal Ganglia / Thalamus, respectively. The color bar represented t-score of the contrast. (B) The ROIs demonstrated significant stronger activity in ‘Listen + Rehearsal’ condition than ‘Resting’ condition, and significant difference between ‘Continuous listen’ and ‘Resting’ conditions. From left to right in the top row are plots for ROIs with their centers in Area Spt, left Premotor Cortex (lPMC), and left Inferior Parietal Lobule (lIPL), from left to right in the bottom row are plots for ROIs with their centers in left Cerebellum (lCB), right Cerebellum (rCB), and right Inferior Parietal Lobule (rIPL), respectively. (C) The ROIs demonstrated significant stronger activity in ‘Continuous listen’ condition than ‘Listen + Rehearsal’ condition. From left to right are plots for ROIs with their centers in left Superior Temporal Gyrus (lSTG), right Superior Temporal Gyrus (rSTG), right posterior Superior Temporal Gyrus (rpSTG), right postcentral gyrus (rPC), and cerebellar vermis (CBV), respectively. The colorbar represents the t-score of the contrast. The plot of each ROI has two insets: a sagittal or axial slice image labeling the spatial location of the ROI in MNI space, in which the green crosshair indicates the location of its center of mass, and a bar plot shows the averaged percentage signal change (PSC) in ‘RS’ and ‘RU’ conditions. For the bar plot, the error bars represent standard error of means (SEM). For statistical significance, ‘*’: FWE corrected p < 0.05; ‘***’: FWE corrected p < 0.005.

The analysis based on the tasks in the ‘localizer’ runs yielded 14 ROIs. The analytical contrasts and conjunctions, the center coordinates in MNI space, the approximate brain region of the centers, and number of voxels for these ROIs are listed in Table 1. Based on the statistics obtained from ROI contrasts, these ROIs could be separated into 3 functional groups. The first group (Fig 2A) consists of three ROIs that activated primarily during covert rehearsal (listen + rehearse vs. listen + rest). These ROIs were centered on the medial frontal cortex, left inferior frontal gyrus, and left putamen in the striatum. More specifically, the clusters of voxels mainly covered the brain regions of the supplementary motor area (SMA) / anterior cingulate cortex (ACC) and extended to the edge of presupplementary motor area (preSMA), Broca’s area (left pars triangularis, pars opercularis, lIFG), and left striatum (lSTR) / basal ganglia and part of the thalamus. The second group of six ROIs was more auditory-motor in their response properties in that they were activated during both listen+rehearsal vs. rest and continuous listening vs. rest. These ROIs were centered on area Spt, a functional area at the posterior extent of the Sylvian fissure at the parietal-temporal boundary (Hickok, Buchsbaum, Humphries, & Muftuler, 2003; Hickok, Okada, & Serences, 2009), left premotor cortex (lPMC), bilateral inferior parietal lobule (lIPL & rIPL), and bilateral superior cerebellum (lCB & rCB) (Fig 2B). The third group (Fig 2C) had five ROIs demonstrating stronger activation in the continuous listen condition than the listen+rehearsal condition. These ROIs were located in bilateral middle to posterior superior temporal regions (lSTG and rSTG) which cover both primary and posterior non-primary auditory cortices, the right post-central gyrus (rPC), and the medial anterior cerebellum / cerebellar vermis (CBV).

**Table 1:** Covert speech repetition activated ROIs.

Analytical Conjunction and ROIs	Approximate Center Region In T-T Atlas	Number of Voxels	Center MNI Coordinates		
			x	y	z
*Listen + Rehearsal > Listen + Rest*					
lSTR	Left Putamen	472	-22	0	4
SMA/ACC	Medial Frontal Gyrus (BA6)	384	-4	6	54
lIFG	Left Inferior Frontal Gyrus (BA44)	210	-56	8	20
*(Listen + Rehearsal > Resting)* *AND* *(Continuous listen > Resting)*					
Spt	Left Insula / Superior Temporal Gyrus (BA13)	22	-54	-40	20
lPMC	Left Precentral Gyrus (BA6)	27	-50	-4	46
lIPL	Left Inferior Parietal Lobule (BA40)	289	-42	-50	42
rIPL	Right Inferior Parietal Lobule (BA40)	23	54	-38	50
lCB	Left Superior Cerebellum (Declive)	62	-26	-62	-28
rCB	Right Superior Cerebellum (Declive)	159	28	-62	-28
*Continuous listen > Listen + Rehearsal*					
lSTG	Left Superior Temporal Gyrus (BA41)	4058	-50	-24	10
rSTG	Right Superior Temporal Gyrus (BA41)	3528	54	-18	8
rpSTG	Right Superior Temporal Gyrus (BA39)	838	48	-58	22
rPC	Right PostCentral Gyrus (BA5)	585	24	-42	64
CBV	Cerebellar Vermis	162	2	-38	-8

The response to the RU vs. RS contrast was examined in each of these ROIs. The response to the RU and RS conditions were not randomly distributed across the three ROI category groups. Two out of three ROIs in Group 1 (motor pattern) responded significantly more during the RS than the RU condition (lIFG and SMA/ACC), showing their sensitivity to the sequential properties of the production task. Another ROI showed evidence of a difference in response to the RS and RU: the left IPL. Notably, area Spt showed no evidence of sequence sensitivity. All ROI-based paired t-test statistics for the ‘RS vs. RU’ contrast are listed in Table 2.

**Table 2:** Statistics of ROI-based contrasts ‘RS’ vs. ‘RU’.

ROI	t-score	Uncorrected p value	FWE corrected p value
SMA/ACC	3.5	0.003	<0.005
lSTR	2.4	0.03	>0.05
lIFG	3.3	0.004	<0.05
Spt	1.4	0.17	>0.05
lPMC	1.9	0.07	>0.05
lIPL	4.6	0.0003	<0.005
rIPL	2.4	0.03	>0.05
lCB	3.1	0.006	>0.05
rCB	2.9	0.01	>0.05
lSTG	1.6	0.1	>0.05
rSTG	1.4	0.18	>0.05
rpSTG	0.3	0.79	>0.05
rPC	1.1	0.3	>0.05
CBV	1.7	0.1	>0.05

#### Correlation with behavioral measurements

No significant correlation was observed between ROI responses and error rates.

## Discussion

Our goal in the present study was to identify the neural networks involved in syllable sequencing. A number of previous studies have identified a broad network including frontal, temporal-parietal, subcortical, and cerebellar regions that supports speech production. A much smaller set of studies have suggested that only a subset of this production network, frontal lobe motor regions, is specifically involved in sequencing, while other studies have questioned even this conclusion (see Introduction). The study reported here first identified the broader network involved in the perception and production of speech and then independently assessed which of the identified regions varied their response depending on whether individual items or sequences of items were produced. More specifically, we used a syllable sequence production task to maximally drive regions involved in sequence and presumably non-sequence aspects of speech production and to define a set of ROIs. We then evaluated activation in these ROIs in a separate scan that explicitly compared syllable sequencing versus individual syllable production to determine which nodes in the broader network were involved in sequencing.

The broad speech network included regions in the frontal, parietal, and temporal lobes as well as the striatum and portions of the cerebellum. This network can be subdivided into three subgroups of regions according to their auditory and/or motor response properties. Subgroup 1 (auditory) consisted of regions that responded to auditory stimulation but not during the motor (covert rehearsal) phase of the task. These were found in bilateral middle-posterior superior temporal gyrus/sulcus (STG/STS). Subgroup 2 (motor) consisted of regions that responded during the motor phase of the task but not during auditory stimulation. These were found in the left inferior frontal gyrus/ventral premotor cortex (lIFG), medial frontal cortex including the supplementary motor area and anterior cingulate cortex (SMA/ACC), and left striatum with the activation center of mass in putamen (lSTR). Subgroup 3 (auditory-motor) consisted of regions that responded to both the auditory and motor phases of the task. These were found in area Spt and bilateral superior cerebellum (CB).

Sensitivity to the production of sequences was found in two of the motor subgroup regions (lIFG and SMA/ACC) consistent with previous observations by Bohland and Guenther [27]. These regions were not specifically implicated in syllable sequencing in the study by Peeva et al. [16] in that activity was not modulated by the manipulation of syllable order, but this negative result may be due to the effects of overt speech feedback or lack of power to detect an effect. The present study utilized a cleaner design for studying syllable sequencing specifically, which was not the primary goal of Peeva et al., in that the present experiment contrasted the covert production of single syllables with the sequenced production of the same syllables. We conclude that the lIFG and SMA/ACC are important nodes in the network involved in syllable sequence production. Work from non-linguistic domains such as perceptual sequence learning [39] and motor planning [40, 41] suggests that this network may not be specific to syllable sequencing.

We also found sensitivity to production of sequences in the left IPL region. Involvement of parietal cortex in motor sequencing is a prevalent phenomenon in visuomotor sequencing and sequence learning paradigms (for a review, see Ashe et al. 2006) including speech sequence reproduction [25] and learning [29]. The interpretation of the parietal lobe involvement is variable, ranging from associating orthographic and phonological components in naming (Bohland et al. 2006; Heim et al., 2012), to storage of phonological representations in working memory (Baddeley, 1986), to representation of the sensory sequencing task framework (Ashe et al. 2006), to attentional/executive function [42]. Our data showed increased activity in left inferior parietal lobule close to supramarginal gyrus during performance of the RS tasks compared to RU conditions consistent with a functional role in sequencing rather than storage of simple phonological elements, but we cannot rule out an attentional role based on current data.

The sequencing network we identified is quite similar to that which has been implicated in working memory [36, 43] and so one might ask whether our experiment simply re-identified this network. Perhaps, but this doesn’t diminish the result. Our paradigm specifically targeted speech sequencing behavior using a tight control condition. If this identifies networks involved in working memory then we suggest that this tells us something important about the role of working memory-related computations in speech planning [24] rather than providing a reason to dismiss the result as “just” working memory. For example, perhaps the capacity of working memory to compute motor plan differences between past and upcoming posture targets places limits on how much sequencing can be carried out without hierarchical chunking of motor plans. Some have even suggested that working memory exists to enable this sort of motor planning [34]. Relatedly, one may wonder whether differences between the RU and RS conditions are due to increased rehearsal in the RS condition. This is unlikely given the rate at which RS items were presented (2 Hz, which is twice the speed of typical working memory span task paradigms) and given that we observed differences between the conditions only in a subset of areas; previous work has reported rehearsal effects in a much broader swath of the network including area Spt [25, 26]. For a broader discussion of the relation between the identified auditory-motor network and phonological working memory see [25, 36, 44].

The anterior insula has been implicated in speech planning [45], somewhat controversially [46–48]. In the present study, we found no evidence of anterior insular involvement in the performance of our listen and covertly repeat task and therefore no evidence for its involvement in syllable sequencing. This null result is consistent with Fedorenko and colleagues who also failed to find strong evidence of the anterior insula’s involvement in speech production [47] as well as with Bohland and Guenther who reported no effect of sequence complexity in this region [27]. However, Segawa et al. report a sequence learning effect in the frontal operculum that extends into the anterior insula [29]. Evidence for the involvement of the anterior insula in motor speech planning thus remains equivocal.

A major question we had in designing this study was whether area Spt was important for coding sequences of syllables. Spt has been shown to exhibit auditory-motor response properties for speech and tonal stimuli [25, 26], has been associated with phonological level speech production deficits in conduction aphasia [44], and has been argued to be a key hub of the state feedback control network for speech [19, 20]. This raises the question of whether Spt is important for sequencing of phonological information at the syllable level. In the present study we replicated previous work showing that Spt activates both during speech perception and (covert) production. However, we found no evidence to indicate that Spt modulates its response as a function of sequencing load. Spt responded no differently during the individual syllable repetition task and the syllable sequence repetition task. This suggests that Spt is involved in coordinating auditory-motor information either on a more fine-grained scale, such as phoneme sequences, which varied equally across our two conditions, and/or over some higher-level information, such as intonational or melodic contours (Hickok, 2016). The fact that Spt activates equally well during perception and vocalization of speech and tonal stimuli [25, 49] is most consistent with the latter.

Both the left posterior IFG (pIFG; pars opercularis) and the SMA/ACC responded more during the production of syllable sequences compared to the same syllables articulated one at a time. Several previous studies have also implicated these regions in sequence processing for both speech and nonspeech stimuli. The SMA has specifically been implicated in coding the ordinal position of a movement in a sequence rather than the movement itself [50]. Conversely, the left pIFG has been proposed as the storage site for a mental syllabary or sequence chunking mechanism that packages motor speech plans into units for efficient articulation [11, 29, 51, 52]. This region is also functionally and anatomically connected to auditory speech regions which play a critical role in speech production [20]. The weight of the available evidence, including the present study, indicates that an important function of the left pIFG is motor planning over fairly broad temporal and/or structural scales, which is necessary for sequencing several syllables.
